# Relationship between Protein Oxidation Biomarkers and Uterine Health in Dairy Cows during the Postpartum Period

**DOI:** 10.3390/antiox8010021

**Published:** 2019-01-14

**Authors:** Gianfranco Gabai, Elisabetta De Luca, Giovanni Miotto, Gianni Zin, Annalisa Stefani, Laura Da Dalt, Antonio Barberio, Pietro Celi

**Affiliations:** 1 Department of Comparative Biomedicine and Food Science, University of Padova, Legnaro, 35020 Padova, Italy; dlelisabetta9@gmail.com (E.D.L.); laura.dadalt@unipd.it (L.D.D.); 2 Via Argine Sinistro 23 Creola di Saccolongo, 35030 Padova, Italy; giannizin61@gmail.com; 3 Department of Molecular Medicine, University of Padova, 35121 Padova, Italy; giovanni.miotto@unipd.it; 4 Proteomics Center, University of Padova and Azienda Ospedaliera di Padova, 35129 Padova, Italy; 5 Istituto Zooprofilattico Sperimentale delle Venezie—Italian Health Authority and Research Organization for Animal Health and Food Safety, Legnaro, 35020 Padova, Italy; astefani@izsvenezie.it (A.S.); abarberio@izsvenezie.it (A.B.); 6 DSM Nutritional Products, 4303 Kaiseraugst, Switzerland; pietro.celi@dsm.com

**Keywords:** dairy cow, puerperium, uterus, clinical outcome, oxidized proteins

## Abstract

High neutrophil (PMN, Polymorphonuclear neutrophil) counts in the endometrium of cows affected by endometritis, suggests the involvement of oxidative stress (OS) among the causes of impaired fertility. Protein oxidation, in particular, advanced oxidation protein products (AOPP), are OS biomarkers linked to PMN activity. To test this hypothesis, the relationship between protein oxidation and uterus health was studied in thirty-eight dairy cows during the puerperium. The animals were found to be cycling, without any signs of disease and pharmacological treatments. PMN count was performed either through a cytobrush or a uterine horn lavage (UHL). Cows were classified into four groups, based on the uterine ultrasonographic characteristics and the PMN percentage in the uterine horns with a higher percentage of high neutrophil horn (HNH). They were classified as: Healthy (H); Subclinical Endometritis (SCE); Grade 1 Endometritis (EM1); and Grade 2 Endometritis (EM2). AOPP and carbonyls were measured in plasma and UHL. UHL samples underwent Western blot analysis to visualize the carbonyl and dityrosine formation. Plasma AOPP were higher (*p* < 0.05) in EM2. AOPP and carbonyl group concentrations were higher in the HNH samples (*p* < 0.05). Protein concentration in the UHL was higher in the EM2 (*p* < 0.05). Carbonyl and dityrosine formation was more intense in EM1 and EM2. Protein oxidation observed in the EM2 suggests the presence of an inflammatory status in the uterus which, if not adequately hindered, could result in low fertility.

## 1. Introduction

A considerable percentage of dairy cows experience some form of uterine infection during the postpartum period [1]. Physiologically, the composition of the uterine microflora changes over time and bacteria are spontaneously cleared [2]. However, the risk of developing clinical endometritis remains elevated, especially in cows that deliver twins or experience stillbirth, dystocia, retained placenta, or metabolic disorders [1]. Moreover, the development of subclinical endometritis can negatively impact fertility if not detected and cured in time [1,3].

Unresolved inflammation can also increase the risk of embryonic mortality, and it is conceivable that if potentially pathogenic microorganisms are retained in the uterus for a long time after parturition or are introduced at the time of artificial insemination (AI) [4], a chronic inflammatory reaction may occur and compromise the embryonic development. Several scientific investigations have revealed that changes in the endometrial gene expression could be found in animals affected by subclinical endometritis. In addition, gene expression patterns of embryos obtained from cows affected by subclinical endometritis were different from those observed in healthy cows [3]. Indeed, secretions from the inflamed endometrium can affect embryonic development in vitro, leading to a reduction in the blastocyst quality [5].

The presence of high neutrophil counts in the endometrium of cows affected by clinical or subclinical endometritis suggests the potential involvement of oxidative stress, among the mechanisms that could compromise fertility. In dairy cows, both oxidants and antioxidants are involved in several reproductive functions, such as the regulation of follicular fluid environment, folliculogenesis, steroidogenesis, corpus luteum function, and luteolysis [6]; when redox balance is disrupted, several pathological conditions can be observed [7]. In particular, in dairy cows oxidative stress and protein oxidation are implicated in the embryonic mortality [8,9].

Neutrophils are a major source of oxidants in the mammalian species. They express the enzyme NADPH oxidase (NO_X_) that, once activated, generates great amounts of superoxide (O^2−^), which is the precursor of hydrogen peroxide (H_2_O_2_) and other reactive oxygen species (ROS). Hydrogen peroxide can react with the enzyme myeloperoxidase (MPO), giving rise to a strong oxidant and short-living intermediate capable of reacting with halides (Cl, Br, NO), and generate highly microbiocidal species, such as the hypochlorous acid (HOCl). HOCl is a potent bactericidal molecule that can also modify extracellular targets, and affects the function of the neighbouring cells [10]. In particular, HOCl attacks proteins and can induce alterations to both the protein backbone and side chains. These structural alterations are often irreversible and lead to alterations of the protein functions [11]. 

Protein oxidation can be monitored by studying the backbone fragmentation and the products of protein side-chain oxidation. The latter are preferable, as they are relatively stable and several assays are available for their detection. The most frequently used biomarker of protein oxidation is the carbonyl assay, which measures protein carbonyl groups. However, advanced oxidation protein products (AOPP) may be considered a more suitable marker of oxidative stress linked to phagocyte activity [11]. Indeed, activated bovine neutrophils give an important contribution to AOPP formation [12].

Inflammation is a very complex response that is activated to cope with agents challenging tissue integrity, and it should be a rapid, intense response devoted to tissue protection and recovery. If not promptly controlled, inflammation may lead to detrimental consequences and a chronic inflammatory state may occur, which in some circumstances is not confined to the originally inflamed tissue, but assumes the connotation of a chronic challenge, for the whole organism. Recently, the term “oxinflammation” has been proposed to describe the vicious circle linking chronic and systemic oxidative stress to mild chronic inflammation, which may lead to a loss of reactivity to induce an adaptive immune response and predisposition to diseases [13]. It is, therefore, worthwhile investigating the relationships existing between inflammation and oxidative stress and, in particular, oxidized proteins, which have considerably long half-lives, and oxidative damage to proteins can affect their functions as receptors, enzymes, transport, or structural proteins, and generate new antigens and provoke immune response [11].

To test the hypothesis that, in cows with uterine inflammation, a condition of oxidative stress may occur and biomarkers of protein oxidation would be elevated, we monitored the outcome of the gynaecological examinations routinely performed at the end of puerperium, in a dairy herd.

## 2. Material and Methods

### 2.1. Herd, Clinical Management, and Biological Samples

This trial was carried out between February and May 2016 in a commercial farm located in North-Eastern Italy. Over the period of the trial, the herd consisted of 921 lactating and 111 dry cows, on average, animals were milked twice a day, and the average milk yield was 29.7 ± 0.7 kg/day. Cows had free access to water and received ad libitum a total mixed-ration, balanced to meet their nutritional requirements (21.4 kg Dry Matter; 1.1 Milk Fodder Unit/kg Dry Matter; Neutral Detergent Fibre: 32.7%, Starch: 25.6%, Gross Protein: 15.7%).

The reproductive management consisted in a clinical check-up performed soon after calving, followed by a thorough gynaecological examination routinely performed between days 20 and 40, postpartum, consisting in an ultrasonographic examination of the reproductive tract. One week after the gynaecological examination, a uterine swab and cytobrush sampling were undertaken to perform, respectively, a microbiological and endometrial cytology evaluation. In a sub-set of subjects (approximatively 5% of the herd), the veterinary team performed the lavage of the uterine horns to more accurately evaluate the endometrial cytology in each uterine horn, as an additional tool for monitoring the herd fertility [3,14]. A blood sample was also collected from the tail vein, using evacuated heparinized tubes for hepatic and metabolic profiling. After day 65 postpartum, subjects without clinically evident gynaecological problems were subjected to an oestrus synchronization protocol. All clinical procedures were carried out by skilled veterinary surgeons.

Thirty-eight cows were enrolled in the trial with the following inclusion criteria: (i) clinical examinations performed within a homogeneous time interval (day 25 ± 3 postpartum); (ii) availability of the clinical report, fluid samples from uterine flushing and plasma samples. In addition, the animals included in the trial were cycling and did not show clinically evident signs of diseases, nor received any pharmacological treatments before the clinical examination and sample withdrawal.

### 2.2. Uterine Ultrasonographic Evaluation

The uterus was examined by trans-rectal ultrasonography (Easi-Scan, BCF Technology, Bellshill, UK), using a 5 MHz linear probe. Cows were classified, based on uterine involution and ultrasonographic characteristics of the fluid content in the uterine lumen, as Clinically Healthy (CH—normal uterus without fluid in the uterine lumen); Grade 1 Endometritis (EM1—few cavities with a diameter less than 1 mm filled by fluids); Grade 2 Endometritis (EM2—cavities with a diameter of 1–5 mm filled by fluids). Animals affected by pyometra were not included in this study.

### 2.3. Uterine Swab and Microbiological Evaluation

Animals were restrained and the perineum area was disinfected with 70% ethanol. Then, a sterile swab (Equivet, Kruuse, Langeskov, Denmark) protected by a sterile infusion pipette and inside a plastic sheath was introduced into the cervix, avoiding vaginal contamination. Inside the cervix, the first layer of protection (plastic sheath) was ruptured and the pipette was then manipulated through the cervix into the uterus. Then, the sterile cotton swab was exposed through the sealed plastic pipette (second layer of protection) to sample uterine secretions. The swab was pulled inside the pipette and removed while the pipette was maintained inside the uterus, to avoid contamination by vaginal fluid. Swabs were transferred to a 15 ml conical sterile polypropylene tube containing Amies agar gel, refrigerated, and transferred to the laboratory, within 4 h, for microbiological analysis.

At the laboratory, samples were submitted to a bacteriological culture for aerobic bacteria detection. Swabs were streaked onto blood and McConkey agar, according to the procedure described by Quinn et al. [15], and incubated at 37 °C for 16–24 h, under aerobic and anaerobic conditions. Agar plates were observed to detect bacterial growth and presumptive identification of colonies was performed, based on colonial characteristic and Gram stain. Genus and species identification were performed by means of biochemical reactions through the micro-method (API system, Biomerieux, Lyon, France). Antimicrobial susceptibility tests were performed on selected isolates by the disk diffusion method, according to the Clinical and Laboratory Standard Institute (CLSI) guideline VET01-A4 [16]. Results were interpreted using the CLSI resistance breakpoints, according to the VET01-S2 guidelines [17]. 

### 2.4. The Cytobrush Technique

Endometrial cytology samples were collected by the cytobrush technique (CB), as described by Kasimanickam et al. [14], using a commercially available device (Cytology Brush; Minitube, Tiefenbach, Germany). Slides for cytologic examination were prepared within 4 h, after sampling. The CB samples were gently rolled onto a clean microscope glass slide to spread the cellular material. Two slides were prepared from each sample.

### 2.5. Uterine Horn Lavage

Uterine horn lavage (UHL) was performed to harvest samples for endometrial cytology analysis [3,14]. Each horn was washed with 30 mL sterile Dulbecco’s phosphate buffered saline (DPBS; Sigma, Milan, Italy), using a sterile Foley catheter with a protection sheet (Minitube, Tiefenbach, Germany). The infused fluid was harvested in a 50 mL falcon tube by gravity. Samples were transferred to the laboratory and centrifuged for 5 min (700× *g*, 5 min, 4 °C) to pellet the cells. A drop of the sediment was spread on the glass slide and air-dried for cytologic evaluation. The lavage liquid was further centrifuged (2500× *g*, 15 min, 4 °C), to remove tissue debris and the supernatant was concentrated by centrifugation (4000× *g*, 30 min, 4 °C), in Amicon Ultra 3k tubes (Millipore, Billerica, MA, USA; membrane cut-off 3000 Dalton). Samples were aliquoted and stored at −80 °C.

### 2.6. Cytologic Evaluation

Air-dried smears obtained from, both, through the cytobrush and form the UHL samples, were stained with a modified Wright-Giemsa stain (Diff-Quik; Fisher Diagnostics, Newark, DE, USA), following to the manufacturer’s instructions. All slides were examined under a light microscope (Olympus AHBT3, Vanox Microscope, Tokyo, Japan), at a magnification of 400× and 1000×, to identify the individual cell types, including the endometrial epithelial, mononuclear, and polymorphonuclear (PMN, neutrophils) cells. For each slide, one skilled observer counted all the cells in five optic fields (60,000 m^2^). PMN cell count was expressed as a percentage and was equal to the number of PMN divided by the number of PMN, mononuclear, and epithelial cells combined [3,14]. In every cow, the uterine horn displaying the higher PMN count was classified as the high neutrophil horn (HNH), while the uterine horn displaying the lower PMN count as the low neutrophil horn (LNH).

### 2.7. Biomarkers of Protein Oxidation

Protein concentrations in plasma and uterine lavage fluids were measured by the BCA method (BCA Protein assay kit; Pierce Biotechnology, Rockford, IL, USA), following the manufacturer instructions. Carbonyl groups and advanced oxidation protein products (AOPP) concentrations were measured in plasma and lavage fluids, as biomarkers of protein oxidation, as previously described [12].

To further characterize the protein oxidation in the uterine secretions, representative samples of the uterine lavage fluid were selected from six subjects, based on the results of the clinical examination and the percentage of neutrophils in the cytobrush (Table 1), and were used to detect carbonylated proteins and dityrosine formation by SDS-PAGE and Western blot analysis.

The uterine lavage fluid samples were diluted 1:1 in 2× Laemnli sample buffer (Sigma-Aldrich, St. Louis, MO, USA), boiled for 5 min at 95 °C, and separated by the NuPAGE electrophoresis system (Invitrogen, Thermo Fisher Scientific, Monza, Italy). Monodimentional SDS-PAGE was performed using Bis-Tris 4–12% polyacrylamide gels (NuPAGE Novex, Invitrogen, Monza, Italy) with an MES Running Buffer at 160 V, for approximately 1 h (until the front line reached the end of the gel). Each gel was run in duplicates—one was used for the Western blot analysis; the other was stained by the EZBlue Staining Reagent (Sigma-Aldrich, St. Louis, MO, USA) and was used to assess the sample conditions. 

For the analysis of protein carbonyls, the samples were derivatized by 2,4-Dinitrophenylhydrazine (DNPH, Sigma-Aldrich, St. Louis, MO, USA) before electrophoresis, as described elsewhere [18]. Following electrophoresis, gels were blotted (350 V, 1 h, 4 °C) onto nitrocellulose membranes (0.45 m; GE Healthcare, Amersham, UK) in a Laemmli transfer buffer (25 mM TRIS-base, 192 mM Glycine and 20% Methanol, pH 8.3), using a trans blot apparatus (Elettrofor, Rovigo, Italy). 

To detect the DNP-derivatized carbonyl groups, the membranes were carefully washed in deionized water and blocked overnight, at 4 °C, with 10% skimmed milk and 0.1% Triton × 100, then they were incubated for 1 h, at room temperature, using a rabbit anti-DNP antibody (Sigma-Aldrich, St. Louis, MO, USA) diluted 1:35,000 in PBS, with 0.1% Triton ×100. Membranes were washed three times for 10 min, with a washing buffer (0.1% Triton × 100 in PBS) and then incubated for 1 h, at room temperature, with a horseradish peroxidase (HRP)-conjugated anti rabbit IgG (1:100,000; BioRad, Hercules, CA, USA).

To detect dityrosine formation, the gels were blotted onto the nitrocellulose membranes and blocked for 3 h, at room temperature, with 10% skimmed milk and 0.05% Tween-20 (Sigma-Aldrich, St. Louis, MO, USA) and then incubated overnight at 4 °C, with a monoclonal mouse anti-dityrosine antibody (Advanced BioDesign, Saint-Priest, France), diluted 1: 1000, in PBS, with 0.05% Tween-20. The membranes were washed three times for 10 min with a washing buffer (0.05% Tween 20 in PBS), and incubated for 2 h, at room temperature, with a horseradish peroxidase (HRP)-conjugated anti mouse IgG (1:8000; BioRad, Hercules, CA, USA).

Finally, the membranes were washed three times with the same washing buffers, and the oxidized proteins were visualized by Immobilon Western Chemiluminescent HRP Substrate (MILLIPORE, Billerica, MA, USA) and exposure to autoradiographic films (GE Healthcare, Amersham, Piscataway, UK). Protein bands in the SDS-PAGE gels and autoradiographic films were scanned, using an ImageScanner apparatus (Amersham Biosciences, Piscataway, NJ, USA) and analyzed by the software ImageMaster (Total Lab, Amersham Biosciences Piscataway, NJ, USA).

### 2.8. Data Analysis

For the diagnosis of subclinical endometritis, a threshold of 5% neutrophil was applied for the cytological evaluation performed on both the CB and UHL samples [3,19]. The agreement between the diagnosis of the subclinical endometritis performed by the evaluation of the CB samples and those performed by the evaluation of UHL samples, obtained from the uterine horns displaying the higher (HNH, High Neutrophil Horn) and the lower (LNH, Low Neutrophil Horn) neutrophil percentage, was measured using Cohens kappa.

The clinical classification was combined with the percentage of neutrophils observed in the HNH after one week. Four groups were obtained: (i) Healthy cows (H, *N* = 8): Clinically healthy subjects with PMN < 5%, one week after the clinical evaluation. (ii) Subclinical Endometritis (SCE, *N* = 18): Clinically healthy subjects and PMN ≥ 5%, one week after the clinical evaluation. (iii) Grade 1 Endometritis (EM1, *N* = 6): Animals clinically diagnosed as EM1 and PMN ≥ 5%, one week after the clinical evaluation. (iv) Grade 2 Endometritis (EM2, *N* = 6): Animals clinically diagnosed as EM2 and PMN ≥ 5%, one week after the clinical evaluation.

The relationship between the detectable presence of bacteria and oxidative stress biomarkers was studied by the Mann-Whitney test. Biomarker differences among the groups were studied by the non-parametric Kruskal-Wallis one-way ANOVA and pairwise multiple comparisons. AOPP and carbonyl group concentrations between HNH and LNH were compared by the Mann-Whitney test. Correlations among the biomarkers were measured in plasma and UHL samples and neutrophil percentages observed in the CB and UHL samples were determined by the Pearson’s correlation coefficient. All statistical tests were performed by the SPSS 24.0 software [20].

## 3. Results

Most cows (37/38) were positive to at least one bacteria genus/species isolated in the microbiological cultivation from the uterine swabs (Table 2).

No effects of bacteria positivity on AOPP and carbonyl group concentrations were observed either in the plasma or the UHL samples. Significant correlations were observed between the CB and LNH (*r* = 0.668, *p* < 0.001) and the CB and HNH (*r* = 0.588, *p* < 0.001) neutrophil percentages. LNH and HNH neutrophil percentages were highly correlated (*r* = 0.857, *p* < 0.001). The diagnosis of subclinical endometritis performed by the CB sample evaluation showed a low degree of agreement with that performed by the HNH sample evaluation (k = 0.296 ± 0.1; T = 2.571; *p* < 0.01), and a moderate degree of agreement with that performed by the LNH sample evaluation (k = 0.485 ± 0.133; T = 3.144; *p* < 0.01).

**Figure 1:**
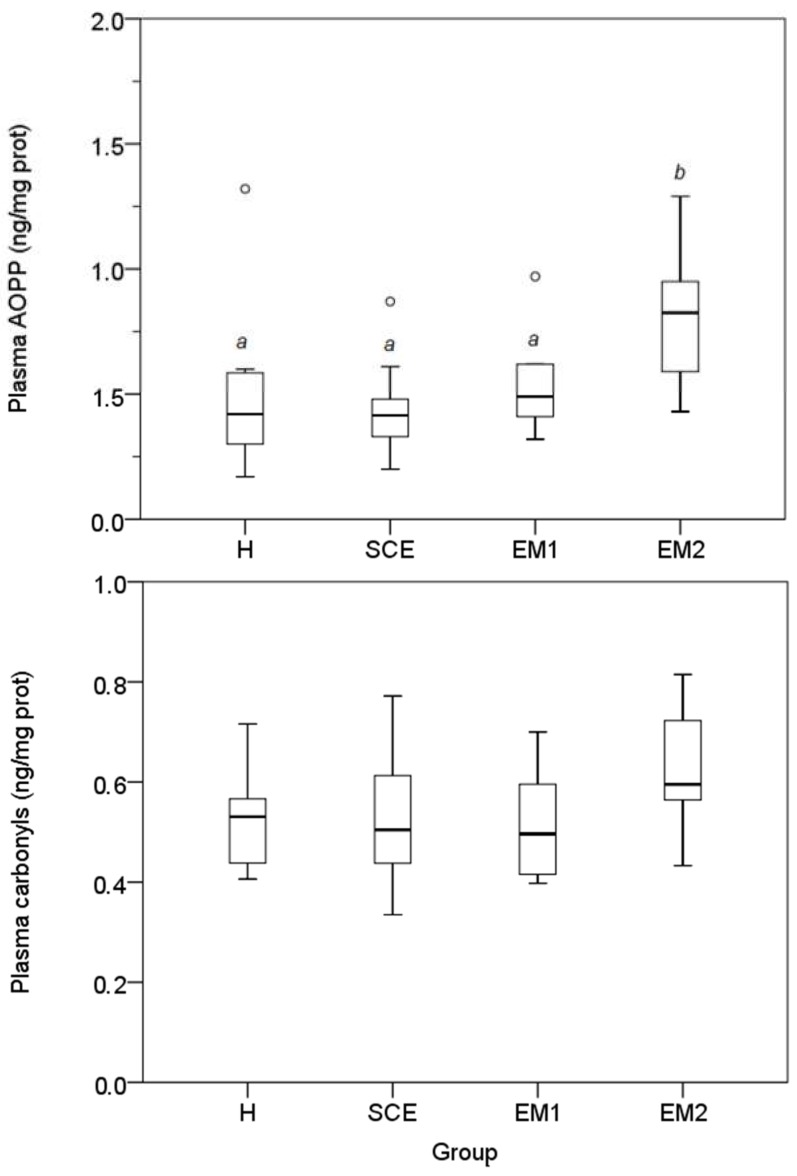
Plasma AOPP and carbonyl group concentrations in the four groups of cows. Different superscripts (^a,b^) indicate statistically significant differences (*p* < 0.05; Kruskal-Wallis one-way ANOVA and pairwise multiple comparisons, SPSS 24.0). Healthy cows (H): Clinically healthy subjects with PMN < 5%, one week after the clinical evaluation. (ii) Subclinical Endometritis (SCE): clinically healthy subjects and PMN ≥ 5%, one week after the clinical evaluation. (iii) Grade 1 Endometritis (EM1): Animals clinically diagnosed as EM1 and PMN ≥ 5%, one week after the clinical evaluation. (iv) Grade 2 Endometritis (EM2): Animals clinically diagnosed as EM2 and PMN ≥ 5% one week after the clinical evaluation.

**Figure 2:**
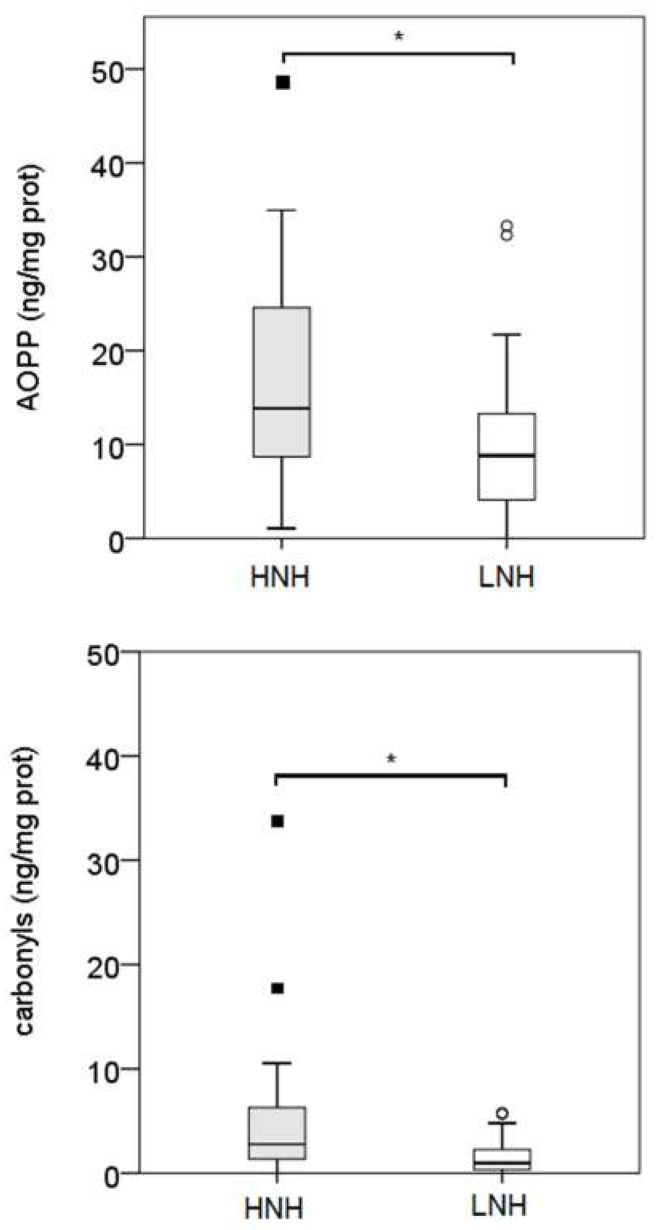
AOPP and carbonyl group concentrations in the uterine horn lavage samples obtained from the uterine horns displaying the higher and lower neutrophil percentages (HNH) and (LNH), respectively. The asterisks (*) indicate statistically significant differences (*p* < 0.05; Mann-Whitney test, SPSS 24.0).

Plasma AOPP concentrations were higher in the EM2 group (*p* < 0.05). Conversely, carbonyl groups concentrations did not show significant differences among the diagnosis groups (Figure 1). A significant correlation was observed between the plasma AOPP and the carbonyl groups (*r* = 0.595; *p* < 0.01). Overall, AOPP and carbonyl group concentrations were higher in the HNH samples (*p* < 0.05; Figure 2). AOPP and carbonyl group concentrations measured in both LNH and HNH were not different among the diagnosis groups. No significant correlations were found between the plasma and the UHL AOPP and carbonyl groups; while high correlations between the LNH and HNH were found in both the AOPP (*r* = 0.752, *p* < 0.001) and the carbonyl group (r = 0.506, *p* < 0.01).

**Figure 3:**
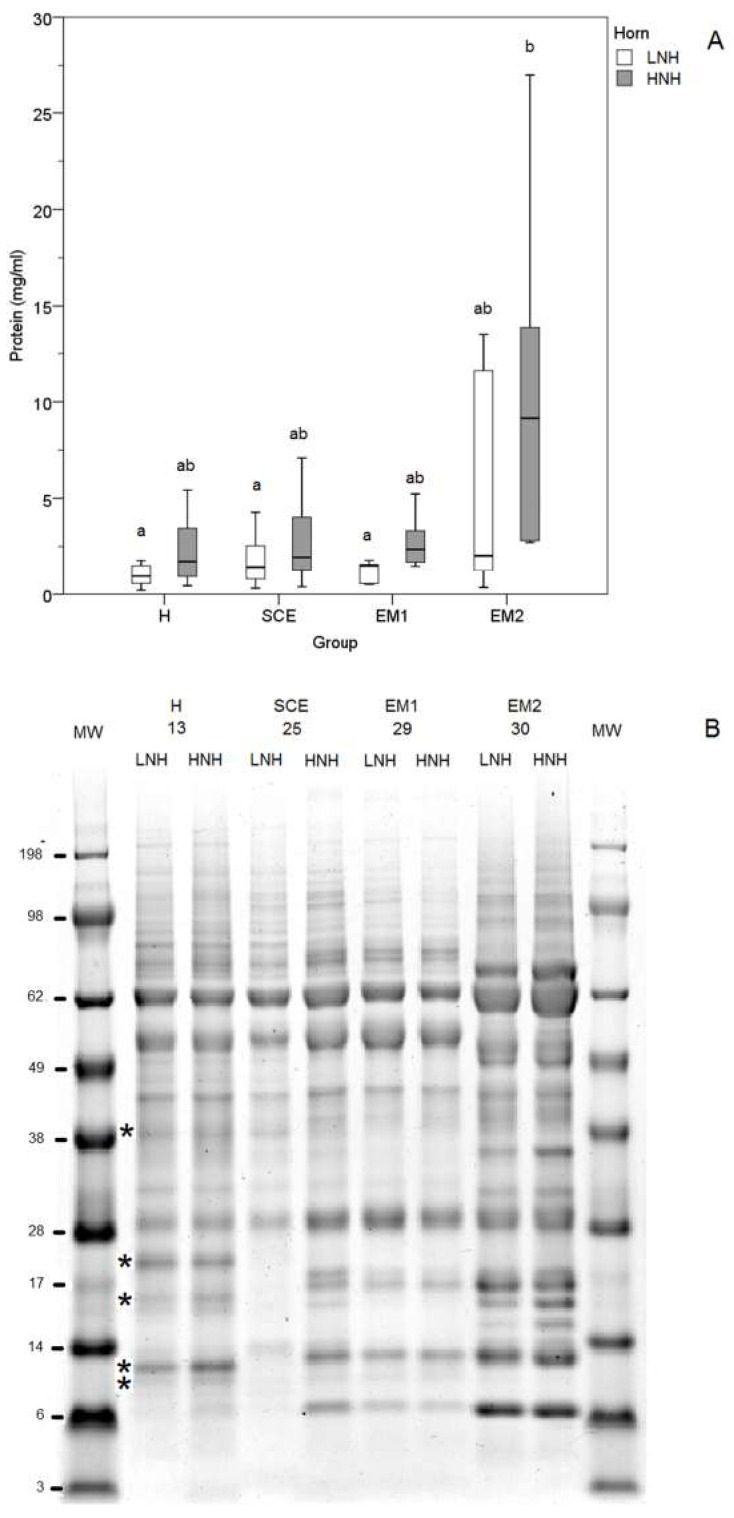
Concentrations (**A**) and SDS-PAG electrophoresis (**B**) of the proteins in the uterine horn lavage (UHL) fluids obtained from the uterine horns with the higher and lower neutrophil percentage (HNH) and (LNH), respectively (**A**). MW: molecular weights; H (Healthy cows): Clinically healthy subjects with PMN < 5%, one week after the clinical evaluation; SCE (Subclinical Endometritis): Clinically healthy subjects and PMN ≥ 5%, one week after the clinical evaluation; EM1 (Grade 1 Endometritis): Animals with grade 1 clinical endometritis and PMN ≥ 5%, one week after the clinical evaluation; EM2 (Grade 2 Endometritis): Animals with grade 2 clinical endometritis and PMN ≥ 5%, one week after the clinical evaluation. Different superscripts (a, b) indicate a statistically significant different group (*p* < 0.05; Kruskal-Wallis one-way ANOVA and multiple comparisons, SPSS 24.0). The asterisks indicates electrophoretic bands exclusively present in the H cows.

Protein concentrations in the UHL samples were significantly higher in the HNH of the EM2 subjects (*p* < 0.05) (Figure 3A), and electrophoretic profiles were different among animals (Figure 3B). In particular, bands at 39, 21, 15, 12, and 10 kDa were observed in the H subjects, which were not detected in the SCE, EM1, and EM2 cows. In the SCE cows, the LNH electrophoretic profile differed from that in the HNH, which was more similar to those observed in the EM1 cows. EM2 cows showed marked differences in the electrophoretic profile, with bands at 68, 18, 16, 15, 14, 13, and 9 kDa suggesting extensive proteolysis (Figure 3B).

**Figure 4:**
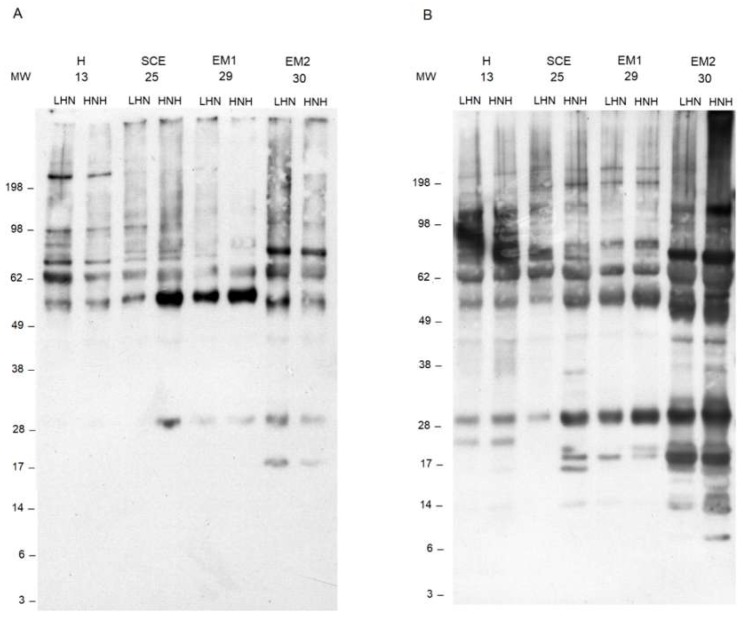
Western blot analyses of carbonyl groups (**A**) and dityrosine (**B**) detected in the uterine horn lavage (UHL) fluids, with the higher and lower neutrophil percentage (HNH) and (LNH), respectively, collected from four representative cows. MW. molecular weights; H (Healthy cows): Clinically healthy subjects with PMN < 5%, one week after the clinical evaluation; SCE (Subclinical Endometritis): Clinically healthy subjects and PMN ≥ 5%, one week after the clinical evaluation; EM1 (Grade 1 Endometritis): Animals with grade 1 clinical endometritis and PMN ≥ 5%, one week after the clinical evaluation; EM2 (Grade 2 Endometritis): Animals with grade 2 clinical endometritis and PMN ≥ 5%, one week after the clinical evaluation.

Carbonyl staining was different among the animals, and the most evident were the following. An intense 72 kDa band was present in the EM2 cows. The bands at 55 kDa were more intense in the HNH of the SCE and in the EM1 subjects. Positive carbonyl staining was observed at 28 and 18 kDa in the HNH of the SCE animals, and in the EM1 and EM2, but not in the H cows (Figure 4A). In general, dityrosine staining was more intense in the EM2 cow, which showed a pronounced laddering between 8 and 30 kDa in the HNH, and an intense staining at 69 kDa. In particular, the EM2 animal showed an intense band at 18 kDa, in both uterine horns. The intensity of the bands observed at 55 and 30 kDa progressively increased from the H to the EM2 animals. Peculiar bands were observed at 22 kDa in the H cows, and at 16, 18, and 20 kDa in the HNH of the SCE cows (Figure 4B).

## 4. Discussion

In this study, we could observe a relationship between uterine inflammation and the biomarkers of protein oxidation. In particular, a mild condition of oxidative stress, characterized by higher AOPP plasma concentrations was observed in the EM2 subjects.

One limitation of this study was the unavoidable time interval between the clinical examination of the enrolled subjects and the collection of biological samples, which made the classification of the cows challenging. As the threshold of the neutrophil proportion for the diagnosis of subclinical endometritis is still under discussion, and the percentage of neutrophils is quite variable during the postpartum period [3], we opted for a more stringent definition of subclinical endometritis and adopted the threshold of 5% [19], to define an inflammatory status. The neutrophil percentage observed in the HNH was always higher than 5%, in both the EM1 and EM2 subjects, suggesting that uterine inflammation was still present between the clinical examination and the UHF collection. Although neutrophil percentages in the HNH and the LNH correlated with those observed in CB, and the diagnosis of subclinical endometritis performed by the CB sample evaluation agreed with that performed by the HNH and the LNH sample evaluation, the classification based on the neutrophil percentage observed in the HNH seemed more accurate, as it allowed the identification of the SCE subjects.

In our study, bacterial contamination was observed in all but one cow, although any relationship between bacterial contamination and either biomarkers of protein oxidation or clinical classification could not be detected. The cow’s uterus is usually contaminated with several bacteria species, but contamination does not mean that the animals will develop a clinical disease [1]. *Escherichia coli* and *Trueperella pyogens* were the only pathogenic bacteria [21] isolated from seven and two (both EM2) cows, respectively. It is generally believed that subclinical endometritis is associated with unspecific infections, while known pathogens are associated with clinical endometritis [3]. In any case, the uterine flora undergoes repeated cycles of contamination and clearance during puerperium [2], and the cows may have been in contact with pathogenic bacteria, in the early stages of postpartum, eventually resulting in an inflammatory reaction. Indeed, unresolved inflammation, potentially pathogenic microorganisms retained in the uterus for a long time after parturition, or introduced at AI [4], may result in a chronic inflammatory reaction. Our observations are in line with those of McDougall et al. [22] and Peter et al. [23], who suggested that endometrial cytology is a better predictor of fertility than intra-uterine bacteriology or vaginal discharge scoring.

We chose to investigate the relationships between uterine health and protein oxidation at the end of puerperium (approximately, three-four weeks after parturition), as the clinical outcome of the uterus at this stage may affect the uterine clinical conditions, as long as day 50 of lactation. Indeed, cows classified as healthy on day 24–30, postpartum, had more chances to remain healthy later in the postpartum; while uteri with clinical or subclinical endometritis tended to remain in an inflammatory status [23]. The same authors [23] observed that the cows did not maintain the same clinical status throughout the postpartum (between days 24–30 and 45–51 postpartum), and the severity of inflammation might differ during the time course of the postpartum, with the more severe reactions observed around day 45–51. The findings of this work could be well integrated in the general hypothesis that the occurrence of endometritis during early postpartum has documented detrimental effects upon the cow’s fertility [1], and add some information about the mechanisms that may explain the long-term effects of early uterine health on the subsequent reproductive activity.

The initial uterine reaction to infections involves the activation of the innate immune system with a massive influx of neutrophils, secretion of antimicrobial peptides [1], and alterations in the endometrial and embryonic gene expression [3]. The intensity of the inflammatory response should match the severity of the insult [21], and the inflammatory response should be rapid and robust to fight against invading pathogens. Once the infection is controlled, the inflammatory reaction should terminate, tissue repairing should quickly take-over and homeostasis regained. Otherwise, if the inflammatory reaction is delayed or inadequate, or the anti-inflammatory feedback mechanisms fail to switch-off the inflammatory process, the infection may not be resolved, and a detrimental over-response may occur, leading to “collateral” damages to the animals’ tissues and, eventually, to chronic inflammation [24,25,26]. It has been hypothesized that the persistence of neutrophils in the uterus may reflect a failure in the innate immune system to return to homeostasis [24].

Neutrophils are a major source of oxidants in the mammalian species, as they generate great amounts of superoxide, hydrogen peroxide, HOCl, and other reactive oxygen species [10]. Although it is debatable if oxidative stress is the cause of a specific disease or it is an epiphenomenon of the disease process, the generation of pro-oxidant chemical species is one of the most evident consequences of inflammation. The production of pro-oxidant species usually begins locally, close to the sites of tissue damage or infection, but it can become chronic, if the inflammatory response is not properly controlled. To describe this low level of inflammation associated with oxidative stress, the term “oxinflammation” has been recently proposed [13].

Oxidative stress may negatively affect the neutrophil regulatory mechanisms. For example, the excessive/prolonged release of superoxide due to the association of the enzyme xanthine oxidase with TLR4 enhances the expression of NF-кB-dependent pro inflammatory cytokines [27]. Additionally, the modality of neutrophil death may be crucial for the evolution of the inflammatory process. Neutrophil cytolysis likely implies the amplification of the inflammatory response [10], and the accumulation of oxidation products at an inflammatory site, such as high amounts of oxidized proteins observed in the present study, may lead to the progressive reduction of neutrophil viability [12] and, perhaps, regulatory properties. This hypothesis is even more attractive if one considers that neutrophils contribute to the regulation of the adaptive immune response, in a number of ways, such as the release of the anti-inflammatory cytokine IL-10 [26]. Interestingly, high levels of IL-10 were observed in cows with subclinical endometritis, which might contribute to the weakening of uterine resistance to pathogens and lead to the persistence of the inflammation, postpartum [28].

Considering the scenario depicted above, the higher plasma AOPP concentrations observed in the EM2 animals, in this study, may depend on a general inflammatory status that activates peripheral neutrophils, and may well be an example of oxinflammation which, if not adequately hindered by the animal’s homeostatic systems, could result in low fertility, later, during postpartum. Indeed, AOPP are considered as biomarkers of neutrophil ROS generation [11,12,29]. Conversely, protein carbonyls are less sensitive biomarkers of MPO and cannot be considered as specific products of the neutrophil oxidative burst and HOCl [11,29], as a variety of oxidation mechanisms can lead to their formation [11,30]. An increase in the carbonyl group formation can be observed, following the incubation of bovine serum albumin (BSA) with bovine neutrophils in the later stages of incubation [12]. Therefore, the lack of a significant relationship between the plasma carbonyl groups and the intensity of the uterine inflammation, was not surprising.

In this study, it was possible to make some observations about the oxidative fate of the proteins contained in the uterine fluids. Protein concentrations tended to be higher in the HNH, in all inflammatory conditions, and were higher in the EM2 cows. On average, both AOPP and the carbonyl groups were higher in the HNH. These findings were expected as they were probably the result of a leakage of plasma proteins through the inflamed endometrium that are possibly related to the severity of inflammation. Once in the uterine horns, the plasma proteins were oxidized by the activated neutrophils. Indeed, electrophoresis and Western blot analyses revealed that proteolysis was more pronounced and carbonyl and dityrosine formation were more intense in the EM1 and EM2 cows, although they were also considerably high in the HNH of the SCE cows. Unidentified bands of positive carbonyls and dityrosine were observed at 30 kDa in EM1, EM2, and in the HNH of the SCE cows. Several candidate proteins related to the immune function that migrate around this molecular weight, can be preferential targets of oxidative modifications, and may contribute to AOPP formation. Alternatively, those bands may represent fragments of larger proteins that have been cleaved by neutrophil proteolytic enzymes and oxidized. Therefore, further studies are needed to identify what proteins are present in those bands, and any attempt to formulate a hypothesis is highly speculative. In this context, it is important to note that, in general, the dityrosine formation was more intense than the protein carbonylation.

Dityrosine formation reflects the activity of the activated neutrophils and monocytes, which uses the H_2_O_2_-MPO enzyme system, in the presence of physiological concentrations of free tyrosine and chlorine, to convert tyrosine into o,o’-dityrosine, through the formation of a highly reactive tyrosyl radical. When the tyrosyl radical meets a protein tyrosyl residue, several end-products may be formed, such as tyrosylated proteins and, eventually, o,o’-dityrosine protein cross-links [31]. Dityrosine is a major component of the AOPP, which are a heterogeneous group of protein products containing also chlorinated amino acid residues, such as 3-chlorotyrosin and 3,5-dichlorotyrosine, pentosidines and carbonyls [32]. Therefore, AOPP may serve as a proxy for the estimation of the dityrosine concentration and the assessment of phagocyte activation.

Some preliminary data about the identification of proteins contained in few electrophoretic bands have been obtained by the ESI-QUAD-TOF mass spectrometry (G. Miotto-unpublished data). This helped in the interpretation of the electrophoretic and Western blot patterns, and allowed some observations about the secretion and the degree of oxidation of proteins in the bovine uterus, during the postpartum inflammation.

The band observed at 55 kDa in all UHF samples was identified as serum albumin by mass spectrometry. In the EM2 cows, serum albumin was extensively oxidized within the uterus, as suggested by the intensity of the bands at 55 kDa, observed in both carbonyl groups and dityrosine Western blot analyses. Oxidized BSA may leak back into the circulation and, once in the blood, it may contribute to an increase in the total circulating AOPP and the recruitment and activation of peripheral neutrophils. In humans, oxidized serum albumin (HSA) can bind and activate peripheral neutrophils [33]. It has been hypothesized that serum proteins that extravasate at the site of inflammation and undergo oxidative alterations induced by neutrophils, may represent a general proinflammatory enhancer mechanism, which attract and direct neutrophils at the center of the inflammation [34]. The protein oxidation mechanisms responsible for the activation of neutrophils may not be univocal. Indeed, Kormoczi et al. [34] speculated that HSA oxidized by H_2_O_2_ was responsible for the neutrophil activation. Additionally, HOCl-treated HSA (chlorinated HSA) was able to cause an oxidative response in human neutrophils [33]. Conversely, chlorinated BSA formed in vitro could not trigger an oxidative response and could even mitigate the generation of free radicals from PMA-stimulated bovine peripheral neutrophils [12]. It is possible that massive accumulation of oxidized proteins at the site of inflammation results in a progressive loss of neutrophil viability that could influence the inflammatory process itself.

The main protein observed at 68 kDa was identified as lactoferrin by mass spectrometry. Lactoferrin is a multifunctional, highly glycosylated, iron-binding protein, secreted in milk, numerous mucosal secretions and by neutrophils [35]. Lactoferrin can be enzymatically cleaved to give origin to several peptides with, among others, antimicrobial and immunoregulatory activities [35], and it is conceivable that this protein contributed to the “laddering” observed in the electrophoretic patterns in the EM2 cows. In addition, bovine lactoferrin has twenty tyrosine residues, four of which (Tyr92, Tyr192, Tyr433, Tyr526) are involved in the two iron-binding sites [36]. Bovine lactoferrin is also rich in proline, arginine, lysine, and threonine [36] that are potential sites of primary protein carbonylation [11]. Finally, bovine lactoferrin is highly glycosylated and contains five potential glycosylation sites and, among these, four are glycosylated [37] and can generate secondary protein carbonylation [11]. Therefore, lactoferrin is a potential substrate for dityrosine and carbonyl formation that, in turn, can alter protein functions.

Other proteins related to neutrophil activation have been identified by mass spectrometry in the molecular weight range between 20 and 10 kDa, where Western blot analyses revealed extensive protein oxidation in the EM1 and EM2 cows, in particular, but also in the HNH of the SCE cows. Bands positive to both carbonyls and dityrosine were observed at 20 kDa, where peptidoglycan recognition protein 1 was identified. No carbonyl positive bands were observed below 18 kDa, while positive dityrosine bands were observed in the molecular weight range between 10 kDa and 18 kDa, where cathelicidin 1 and 4 precursors (respectively, 16 kDa and 18 kDa), protein S100 A9 (17 kDa) and protein S100 A12 (10 kDa) were identified. It is not possible, however, to establish whether or not these identified proteins contributed to the dityrosine formation.

The antibody used to detect dityrosine was raised against 3-(p-hydroxyphenyl) propionic acid dimer conjugated with keyhole limpet hemocyanin (KLH) and recognized dityrosine-protein conjugates, but not the dityrosine protein cross-links. For this reason, we could not identify actual protein irreversible cross-links, although it is conceivable that the cross-link formation did occur. It is worth noting that tyrosine residues have often a functional role in proteins and are hidden within the protein tertiary structure. Therefore, it is plausible that dityrosine formation occurred mainly in the highly denatured or fragmented proteins, in which the tyrosine residues were exposed. As a consequence, the dityrosine formation impaired the protein functions, and these dityrosine-protein conjugates could be immunogenic.

## 5. Conclusion

In conclusion, our data suggested that activated neutrophils extensively oxidize proteins in uterine secretions, in both SCE and EM postpartum cows. A mild condition of oxidative stress characterized by a higher AOPP plasma concentrations can be observed in the more critical inflammatory situations (EM2). Data from this study, however, do not give information about the long-term effects of this mild oxinflammatory condition on fertility. Future research should address the nature and biological role of protein oxidation, and whether focused nutritional interventions can contribute to the control of oxinflammation.

## Figures and Tables

**Table 1:** Characteristics of the animals selected to investigate the oxidation of the proteins present in the uterine lavage fluids by SDS-PAGE and Western blot analysis to detect carbonylated proteins and dityrosine formation.

Clinical Classification	Animal Identification	Neutrophil (%)			AOPP (ng/mg Protein)		Carbonyl Group (ng/mg Protein)	
		CB	LNH	HNH	LNH	HNH	LNH	HNH
H	13	0.1	0.2	1.7	3.7	8.2	0.3	0.7
	15	0.3	1.0	4.5	12.9	24.5	nd	0.3
SCE	3	29.0	17.7	19.5	11.3	16.5	5.8	7.8
	17	5.5	2.3	48.1	8.6	32.4	4.8	34.2
EM1	19	18.0	65.0	62.2	16.4	22.1	2.5	3.2
	29	63.9	78.9	88.5	3.0	3.0	0.3	1.7
EM2	30	39.4	49.9	52.1	9.8	11.9	5.7	6.6
	37	5.1	17.4	24.8	2.4	4.0	nd	0.8

H: Healthy; SCE: Subclinical Endometritis; EM1: Grade 1 Endometritis; EM2: Grade 2 Endometritis; CB: cytobrush; HNH: High Neutrophil Horn; LNH: Low Neutrophil Horn; nd: not detected.

**Table 2:** Positive cows (N) in each diagnosis group to the bacterial genus/species isolated in microbiological cultivation from the uterine swabs of thirty-eight cows. Two or more bacterial genus/species might be isolated and typed from the same cow.

Bacterial Genus/Species	H (*N* = 8)	SCE (*N* = 18)	EM1 (*N* = 6)	EM2 (*N* = 6)
*Staphylococcus* spp.	0	3	0	0
*Staphylococcus xilosus*	1	1	0	0
*Staphylococcus sciuri*	3	2	0	0
*Staphylococcus simulans*	0	2	0	0
*Staphylococcus chromogenes*	2	1	2	0
*Staphylococcus haemolitucus*	0	0	0	1
*Staphylococcus epidermidis*	0	0	0	1
*Staphylococcus warneri*	0	0	0	1
*Bacillus* spp.	1	8	2	4
*Escherichia coli*	2	4	0	1
*Micrococcus luteus*	0	3	0	0
*Leuconostoc*	1	1	0	0
*Citobacter freundii*	0	1	0	0
*Enterococcus*	2	6	0	3
*Aerococcus viridans*	0	2	1	0
*Trueperella pyogenes*	0	0	0	2
*Proteus*	0	1	0	0
*Aerococcus urinae*	0	0	2	1
*Pasteurella*	0	0	0	1
Polymicrobism	7	18	6	6

Healthy cows (H): Clinically healthy subjects with PMN < 5%, one week after the clinical evaluation. Subclinical Endometritis (SCE): Clinically healthy subjects and PMN ≥ 5%, one week after the clinical evaluation. Grade 1 Endometritis (EM1): Animals clinically diagnosed as EM1 and PMN ≥ 5%, one week after the clinical evaluation. Grade 2 Endometritis (EM2): Animals clinically diagnosed as EM2 and PMN ≥ 5%, one week after the clinical evaluation.
